# Black bone and CT-like MRI-based delineation of fracture-prone regions in oral and maxillofacial trauma

**DOI:** 10.1007/s10006-025-01431-6

**Published:** 2025-07-28

**Authors:** Adib Al-Haj Husain, Peter Kessler, Suen An Nynke Lie, Samuel Drack, Egon Burian, Sameena Sandhu, Maximilian Eberhard Hermann Wagner, Bernd Stadlinger, Thomas Frauenfelder, Giovanni Colacicco, Rubens Spin-Neto, Harald Essig

**Affiliations:** 1 Department of Cranio-Maxillofacial and Oral Surgery, University Hospital Zurich, University of Zurich, Rämistrasse 100, 8091 Zurich, Switzerland; 2 Clinic of Cranio-Maxillofacial and Oral Surgery, Center of Dental Medicine, University of Zurich, Zurich, Switzerland; 3 Department of Cranio-Maxillofacial Surgery, GROW School for Oncology and Reproduction, Maastricht University Medical Centre, Maastricht, The Netherlands; 4 Department of Neuroradiology, Clinical Neuroscience Center, University Hospital Zurich, University of Zurich, Zurich, Switzerland; 5 Diagnostic and Interventional Radiology, University Hospital Zurich, University of Zurich, Zurich, Switzerland; 6 Institute of Anatomy, University of Zurich, Zurich, Switzerland; 7 Oral Radiology, Department of Dentistry and Oral Health, Aarhus University, Aarhus C, Denmark

**Keywords:** Magnetic resonance imaging, Black bone, CT-like MRI, Maxillofacial injuries, Maxillofacial surgery, Diagnostic imaging

## Abstract

**Purpose:**

To assess the effectiveness and feasibility of MRI-based delineation of fracture-prone regions in the oral and maxillofacial region using Black Bone and CT-like MRI protocols optimized for dentomaxillofacial imaging with a dedicated 15-channel mandibular coil.

**Methods:**

In this prospective study, healthy volunteers underwent 3T MRI using five protocols: DESS, SPACE STIR, SPACE SPAIR, T1-VIBE Dixon, and UTE. Eight trauma-prone regions, including the nasal septum, orbit, naso-orbito-ethmoidal (NOE) complex, zygomaticomaxillary complex, Le Fort regions, mandible, condyle, and dentoalveolar complex, were assessed. Image quality, artifacts, anatomical delineation, and bone-to-soft-tissue contrast were rated on a five-point Likert scale by three independent observers. Descriptive statistics and inter-rater agreement (intraclass correlation coefficients (ICCs)) were calculated.

**Results:**

Sixteen participants (37.2 ± 12.9 years; 12 males, 4 females) were included, resulting in 80 MRI volumes and 640 regions for evaluation. UTE and VIBE-DIXON sequences achieved the highest ratings for image quality, artifact susceptibility, and anatomical delineation across most fracture-prone regions (ICC: 0.793–1; all *p* < 0.001). UTE excelled in visualizing NOE and Le Fort regions, while VIBE-DIXON performed best in mandibular and orbital imaging. Bone-to-soft-tissue contrast was highest in UTE and VIBE-DIXON, highlighting their diagnostic potential in simultaneous soft and hard tissue imaging. Inter-rater agreement was consistently high (ICC: 0.772–0.976; all *p* < 0.001).

**Conclusion:**

Dedicated trauma-specific MRI protocols show promising potential as a radiation-free modality for maxillofacial trauma imaging, particularly in young adults and pediatric patients. The strengths of each protocol highlight the need for tailored sequence selection to optimize diagnostic accuracy and personalized care.

**Trial registration number**: Swiss National Clinical Trials Portal: SNCTP000005246.

## Introduction

Oral and maxillofacial trauma is a prevalent emergency presentation that can vary in severity and complexity, ranging from isolated avulsed teeth to complex panfacial injuries in polytrauma cases. This variability arises from a wide range of causes and injury mechanisms, with the most common being road traffic incidents, interpersonal physical violence, falls, or injuries from sports activity, predominantly affecting young male adults [1, 2].

Despite the advances achieved in modern comprehensive trauma management, severe maxillofacial injuries can pose a challenge to the timely management of trauma patients due to the close anatomical relationship to the central nervous system and upper airway [3]. Therefore, clinical examination is commonly complemented with three-dimensional X-ray-based imaging modalities, such as computed tomography (CT) or cone-beam computed tomography (CBCT), to uncover hidden high-risk injuries, accurately localize fractures, and assess soft and hard tissue involvement in the facial and oral region, ensuring accurate diagnosis and subsequent effective treatment planning [4]. Despite the benefits of CT, including widespread availability, fast acquisition, comparatively low cost, and excellent capacity for preoperative virtual surgical planning, there are inherent limitations to its use as the diagnostic reference standard [5]. These include limited soft tissue contrast, radiation exposure, and susceptibility to metal artifacts from dental restorations, which can compromise image quality and impair diagnostic accuracy [6].

The long-term adverse health effects of cumulative radiation exposure, including the increased risk of radiation-induced cancer from repeated CT scans, are well known and continuously debated, especially among the younger cohort in trauma settings who frequently require multiple perioperative imaging procedures [7, 8]. Given the current efforts to align maxillofacial trauma workflows with the ALADA (as low as diagnostically achievable) principle, investigations have explored the potential of low-dose protocols and the feasibility of minimizing or even eliminating radiation exposure [9, 10].

Radiation-free magnetic resonance imaging (MRI), primarily used for assessing soft-tissue involvement [11], has advanced significantly in recent years with the introduction of novel dedicated dentomaxillofacial coils and optimized MR protocols for imaging the oral and maxillofacial complex, applicable to both high- and low-field scanners [12–15]. Notably, “Black Bone” and CT-like MRI techniques have overcome the limitations of conventional MRI in allowing the visualization of osseous patho-anatomy while simultaneously preserving good soft-to-bone tissue contrast within short acquisition times [16, 17]. While Black Bone MRI, a gradient-echo-based sequence, enables the differentiation between the black bone and adjacent soft tissues [16], CT-like MRI utilizes ultra-short echo time (UTE), zero echo time (ZTE), or T1-weighted gradient echo (GRE) techniques to generate high-contrast bone images comparable to conventional CT [18]. Feasibility studies in oral and maxillofacial trauma have demonstrated the reliable assessment of surgically relevant preoperative parameters, including accurate delineation of fractures, evaluation of the severity of dislocation, and identification of associated soft tissue involvement [19–22].

This prospective comparative study aimed to evaluate the feasibility and diagnostic performance of MRI-based delineation of fracture-prone regions in oral and maxillofacial trauma using five Black Bone and CT-like MRI protocols specifically optimized for dentomaxillofacial imaging with an advanced 15-channel mandibular coil.

## Materials and methods

### Ethics and study design

Ethical approval was granted by the Cantonal Ethics Commission in Zurich, Switzerland (2022-D0090). All voluntary participants provided written informed consent to participate in this study in accordance with the Declaration of Helsinki and its subsequent revisions and allowed that their images would be used anonymized in publications.

This prospective study recruited healthy volunteers from the Center of Dental Medicine and the University Hospital Zurich, Department of Cranio-Maxillofacial and Oral Surgery, during annual routine examinations between May and December 2022. Inclusion criteria were: (1) age over 18 years and (2) absence of clinical symptoms related to head and neck pathologies. Exclusion criteria included: (1) history of surgical procedures in the past three months (2), acute infections in the maxillofacial region (3), trigeminal nerve damage (4), pregnancy, and (5) standard contraindications to MR imaging. Participants in this study underwent MRI examinations conducted by trained clinical staff and research personnel.

### Scan acquisition and reconstruction

All recruited individuals underwent 3 T MRI scans using a MAGNETOM Skyra system (release VE11E, Siemens Healthineers, Erlangen, Germany). The imaging protocols included gradient specifications of 45 mT/m and 200 T/m/s, employing a dedicated 15-channel mandibular coil (NORAS MRI Products, Hoechberg, Germany). This coil is specifically designed for high-resolution dentomaxillofacial imaging, offering a field of view of 32 × 16 × 16 cm. It features a 14 + 1 receiver coil array integrated into a positioning system optimized for detailed anatomical visualization. The curved phased array coil measures 12 × 38 cm^2^ and incorporates 14 elements arranged between two support bars. Built-in fixation components ensure precise head positioning, while designated openings are provided for the nose and mouth. The central section between these openings is aligned above the upper lip to maintain optimal coil placement. Additionally, the outer wings are flexible, allowing for customized adaptation to the patient’s anatomy. To enhance patient comfort and minimize motion artifacts, optional head fixation, and a mirror system can be employed, which is particularly beneficial for individuals prone to claustrophobia [23].

In-house specifically for dentomaxillofacial imaging optimized Black Bone and CT-like MRI protocols were acquired with sub-millimeter isotropic resolution. The following five protocols were acquired: 3D double-echo steady-state (DESS), 3D fast spin echo short-tau inversion recovery (SPACE STIR), 3D fast spin echo spectral attenuated inversion recovery (SPACE SPAIR), 3D volumetric interpolated breath-hold examination (T1-VIBE-Dixon), and a 3D ultrashort echo time (UTE) prototype protocol. To reduce potential bias related to imaging order and patient fatigue, the acquisition order of MRI protocols was systematically randomized across participants, ensuring that no single protocol was consistently performed at the end of the examination. The sequence parameters were as follows: DESS: repetition time, 11.16 ms; echo time, 4.21 ms; flip angle, 30 degrees; bandwidth, 355 Hz/Px; fat suppression, water excitation normal; Phase encoding direction, R » L; Matrix read/phase 320 × 320; total acceleration factor, off; voxel size (acquisition), 0.38 × 0.38 × 0.75 mm^3^; acquisition time, 12:24 min, SPACE STIR: repetition time, 3300 ms; echo time, 113 ms; flip angle, T2 var; bandwidth, 425 Hz/Px; fat suppression, none; Phase encoding direction, A » P; Matrix read/phase 256 × 256; total acceleration factor, 4; voxel size (acquisition), 0.37 × 0.37 × 0.75 mm^3^; acquisition time, 12:36 min, SPACE-SPAIR: repetition time, 3300 ms; echo time, 115 ms; flip angle, T2 var; bandwidth, 425 Hz/Px; fat suppression, SPAIR, strong; Phase encoding direction, A » P; Matrix read/phase 256 × 256; total acceleration factor, 4; voxel size (acquisition), 0.37 × 0.37 × 0.75 mm^3^; acquisition time, 12:36 min, VIBE-DIXON: repetition time, 5.81 ms; echo time, 2.46/3.69 ms; flip angle, 11 degrees; bandwidth, 660/700 Hz/Px; fat suppression, DIXON, optimal in phase; Phase encoding direction, F » H; Matrix read/phase 380 × 380; total acceleration factor, off; voxel size (acquisition), 0.8 × 0.8 × 1.0 mm^3^; acquisition time, 5:28 min, and UTE: repetition time, 4.62 ms; echo time, 0.04 ms; flip angle, 5 degrees; bandwidth, 1184 Hz/Px; fat suppression, none; Phase encoding direction, A » P; Matrix read/phase 384 × 384; total acceleration factor, 0; voxel size (acquisition), 0.6 × 0.6 × 0.6 mm^3^; acquisition time, 3:07 min. Data acquisition was conducted in an axial or coronal orientation and subsequently reformatted using multiplanar reconstruction for additional plane orientations. The same MRI protocols were also investigated in an earlier study [24].

### Image analysis

MRI data were stored in DICOM format and assessed through the local Picture Archiving and Communication System (PACS) in DeepUnity Diagnost (release v.1.1.1.2, Dedalus HealthCare, Bonn, Germany). The three observers had varying experiences in dentomaxillofacial radiology and different medical specializations: Observer A (S.D.) is a resident in the Department of Cranio-Maxillofacial and Oral Surgery with 1 year of experience; Observer B (A.A.H.) is a resident in the same department with 5 years of experience; and Observer C (E.B.) is a senior physician, board-certified radiologist, and dentist at the Institute of Diagnostic and Interventional Radiology with 10 years of radiological experience. A calibration session between the observers took place with the principal investigator (A.A.H.) to standardize the rating method and reduce potential inconsistencies. To maintain consistency in image evaluation, standardized viewing conditions were implemented. Furthermore, each observer had access to windowing and zoom functions, enabling them to adjust these settings according to their individual preference. Additionally, to ensure objective and unbiased scoring, all observers were blinded to each other’s readouts and the MR protocol and scored all scans in a randomized order.

The qualitative evaluation focused on MRI-based delineation of fracture-prone regions of prevalent types in oral and maxillofacial trauma [25], including nasoseptal injuries, orbital fractures, naso-orbito-ethmoidal (NOE) injuries, zygomaticomaxillary fractures, Le Fort injuries, and mandibular fractures with a particular emphasis on condylar trauma involving the temporomandibular joint (TMJ), and dental trauma. All anatomically relevant structures were assessed using established 5-point Likert scales (described below) to evaluate protocol-specific technical image quality, artifact presence, anatomical delineation, and bone-to-soft tissue contrast.

Technical image quality and artifacts, assessing diagnostic performance, background noise, and resolution, were evaluated using the 5-point Likert scale according to Burian et al. [20] established for MRI-based oral and maxillofacial trauma assessment: 5, excellent, no restrictions for clinical use; 4, very good, containing no substantial adverse effect for clinical use; 3, average, borderline clinical use due to the image quality; 2, poor, substantial adverse effect for clinical use; 1, very poor, images not suitable for clinical use.

Anatomical delineation was evaluated using a modified 5-point Likert scale based on Sabarudin et al. [26]: 5, excellent, appropriate coverage for clinical application with fine details fully visible, providing excellent diagnostic interpretability; 4, good, relevant coverage for clinical needs, with small details visible and good diagnostic interpretability; 3, limited, coverage is present but insufficient; only broad details are visible, affecting diagnostic interpretability; 2, inadequate, coverage is inappropriate and clinically irrelevant, with significant structures not visible, which hinders diagnostic interpretation; 1, non-diagnostic, no structures are visible, and diagnostic interpretation is not possible.

Bone-to-soft-tissue contrast was assessed using the 5-point Likert scale established by Feurriegel et al. [21] for oral and maxillofacial trauma settings: 5, excellent; 4, good; 3, fair; 2, below average; 1, poor.

### Statistical analysis

Descriptive statistics were employed to analyze the qualitative data on technical image quality and artifact susceptibility, anatomical delineation, and bone-to-soft tissue contrast. This included calculation of the median, interquartile range (IQR), minimum, maximum, and frequency distribution of Likert scale scores.

The agreement among observers was assessed to ensure consistency in evaluating the qualitative variables. Inter-rater reliability for the parameters was determined and reported by a two-way random effects model, as indicated in the intraclass correlation coefficient (ICC) type 2:1 analysis, along with the 95% confidence interval. The ICC values were interpreted as follows: excellent (> 0.9), good (0.75–0.9), moderate (0.5–0.75), and poor (< 0.5) [27].

All statistical analyses were conducted with a two-sided significance level of 0.05 using IBM SPSS Statistics software (version 29.0, IBM Chicago, IL, USA).

## Results

Sixteen participants (mean age: 37.2 ± 12.9 years; 12 males, 4 females) were included in this study. This resulted in 80 MRI protocol evaluations per observer, assessing eight fracture-prone regions per protocol, representative of common oral and maxillofacial trauma patterns.

Among the five protocols assessed, the UTE and VIBE-DIXON sequences consistently received the highest ratings for overall technical image quality and artifact susceptibility. UTE achieved an average median score of 5.0 (IQR: 0; range: 2–5), followed by VIBE-DIXON MRI with a median score of 4.5 (IQR: 1.0; range: 2–5). The DESS sequence demonstrated limited to good performance, whereas STIR and SPAIR performed similarly with lower overall ratings. Inter-rater agreement was rated as good to excellent across all five protocols, with ICCs ranging from 0.893 to 0.953 (all *p* < 0.001).

**Fig. 1:**
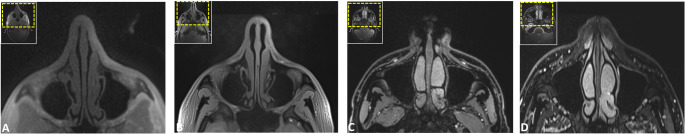
Axial reconstructions illustrating the delineation of the nasoseptal region, emphasizing the differences in bone-to-soft tissue contrast and anatomical delineation of osseous, cartilaginous, and soft tissues across the following protocols: gradient-echo based ultrashort echo time (UTE) (**A**), T1-weighted volumetric interpolated breath-hold examination (VIBE) (**B**), T2-weighted 3D double-echo steady-state (DESS) (**C**), and T2-weighted fast spin echo short-tau inversion recovery (SPACE STIR) (**D**)

**Fig. 2:**
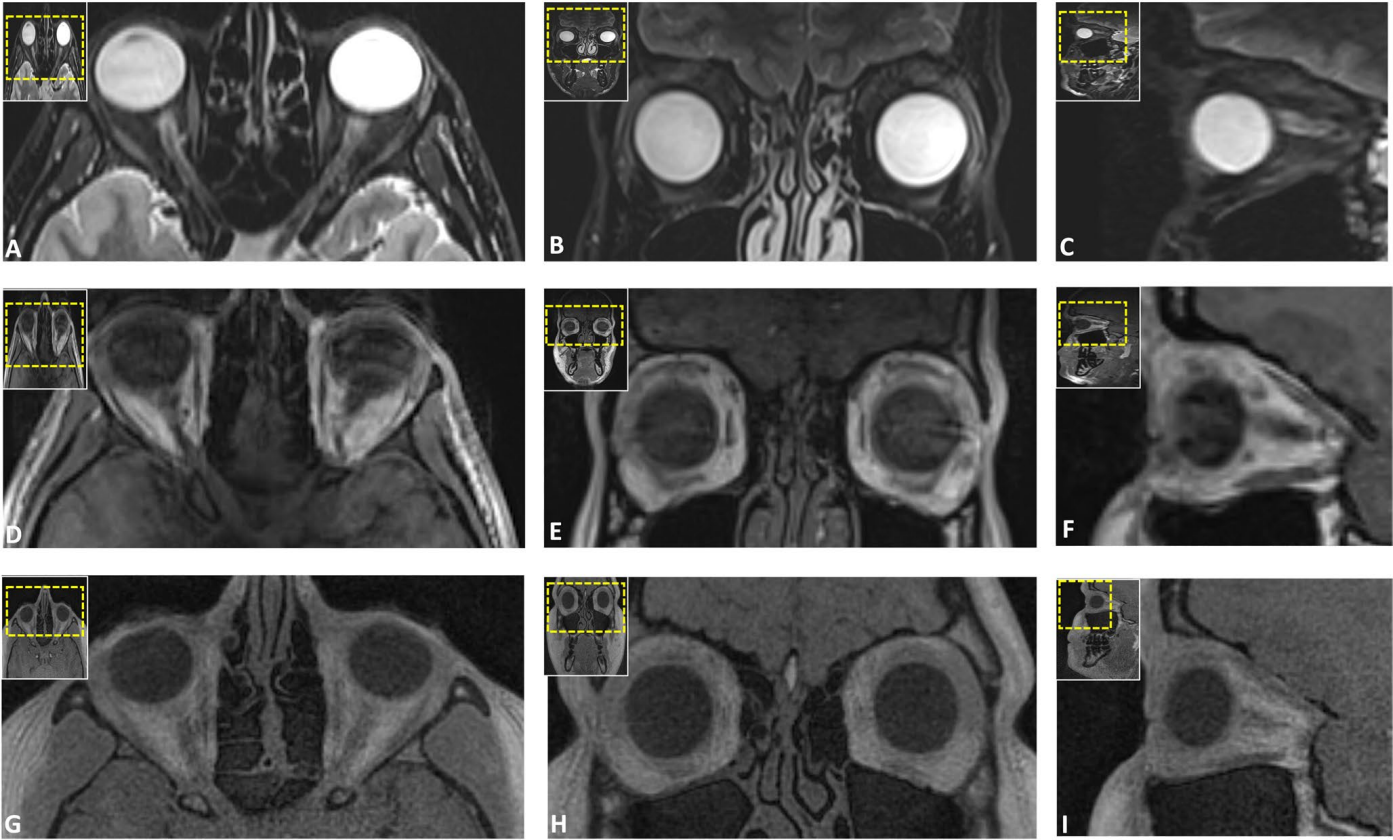
Axial (**A**, **D**, **G**), coronal (**B**, **E**, **H**), and sagittal (**C**, **F**, **I**) reconstructions of the orbital region, highlighting relevant trauma-prone osseous and soft tissue structures, including the orbital walls, optic nerve, and extraocular muscles. Images were acquired using the fast spin echo short-tau inversion recovery (SPACE STIR) (**A**-**C**), volumetric interpolated breath-hold examination (VIBE-DIXON) (**D**-**F**), and an ultrashort echo time (UTE) prototype (**G**-**I**) sequence

**Fig. 3:**
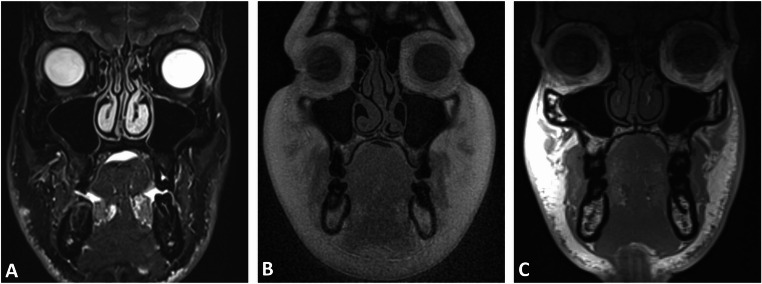
Coronal reconstructions of the fast spin echo short-tau inversion recovery (SPACE STIR) (**A**), ultrashort echo time (UTE) prototype (**C**), and volumetric interpolated breath-hold examination (VIBE-DIXON) (**B**) sequences, illustrating the anatomical depiction of potential midface involvement, including the nasal pyramid, medial orbital walls, and ethmoid air cells in naso-orbito-ethmoidal injuries

Anatomical delineation consistently demonstrated superior performance for the VIBE-DIXON and UTE protocols, with median scores frequently reaching the excellent to maximum values ([4]– [5]) and narrow IQRs, while simultaneously indicating robust reproducibility with high inter-rater agreement across all regions (ICCs: 0.793–0.953; all *p* < 0.001). Evaluation of the frequency distribution of visual grading using Likert scores revealed that UTE-MRI yielded the highest ratings for the NOE region (median: 4.67; IQR: 0; range: 2–5) and fracture-prone Le Fort regions (median: 5.0; IQR: 0.33; range: 2–5), outperforming all other sequences. VIBE-DIXON protocols demonstrated superior visualization of the nasoseptal, orbital, and particularly the mandibular regions, while UTE-MRI, despite slightly lower diagnostic performance in these areas, still achieved a consistently good to excellent rating distribution across the same anatomical regions (Figs. 1, 2 and 3). DESS provided the highest anatomical delineation of the TMJ, particularly in the fracture-prone condylar and subcondylar regions, with a median score of 5.0 (IQR: 0; range: 1.33–5), indicating excellent diagnostic performance. For dental trauma delineation, both DESS and UTE protocols showed the most promising diagnostic potential. In contrast, the SPACE sequences (STIR and SPAIR) demonstrated moderate delineation, with greater variability across anatomical regions and observers, frequently limited by lower contrast and more pronounced artifacts.

Bone-to-soft-tissue contrast was rated highest with UTE and VIBE-DIXON sequences across most regions, achieving median scores between 4 and 5 with narrow IQRs, reflecting excellent contrast and moderate to excellent inter-rater agreement (ICCs: 0.722–0.976; all *p* < 0.001). In contrast, DESS, STIR, and SPAIR sequences demonstrated lower contrast values (median range: 2–3), particularly in complex anatomical regions, indicating limitations in differentiating osseous from adjacent soft tissues.

**Fig. 4:**
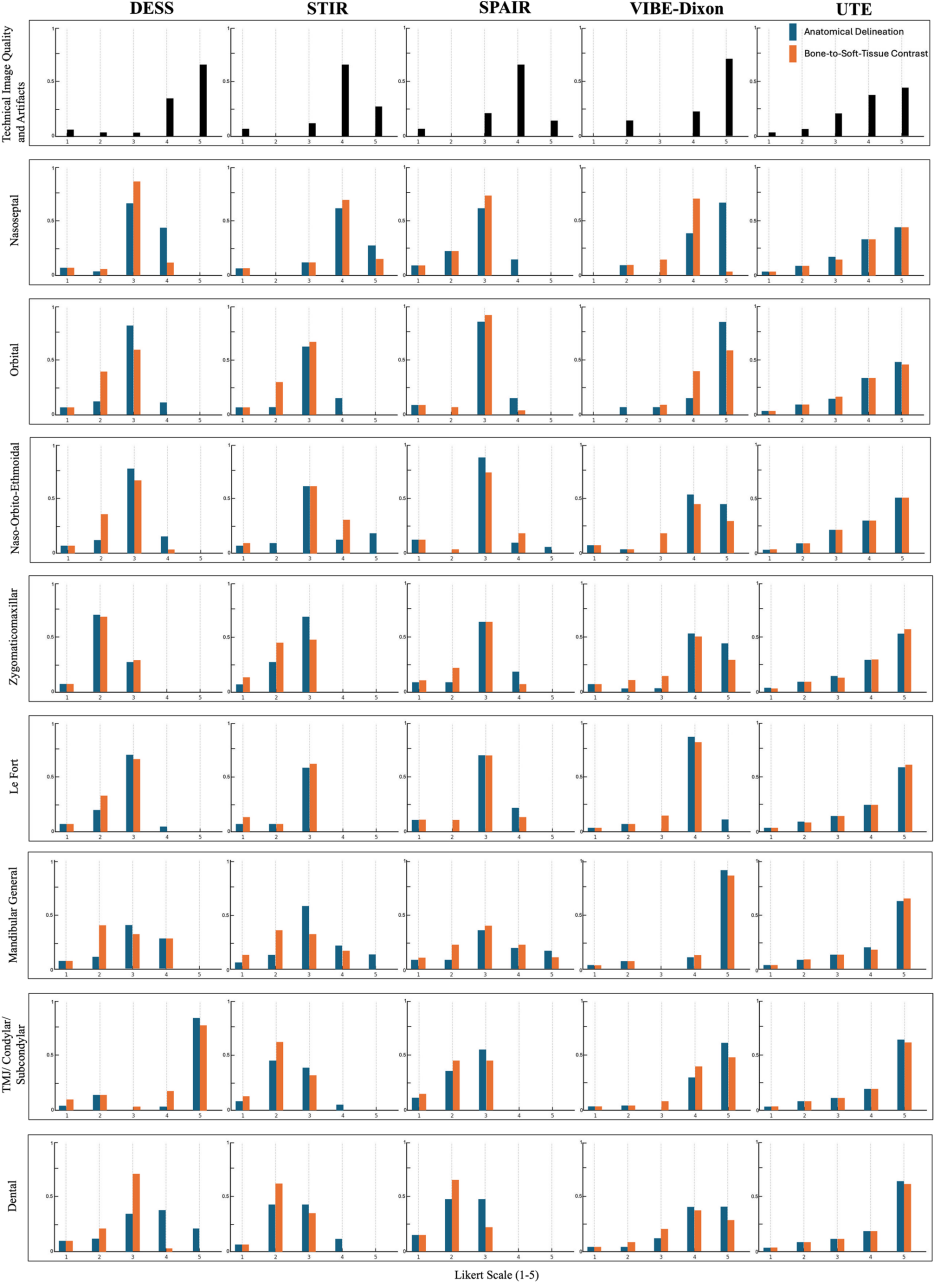
Frequency distributions of ordinal Likert-scale ratings from visual assessments were analyzed across all three observers for each MRI protocol and anatomical region, focusing on anatomical delineation and bone-to-soft-tissue contrast

Detailed information on anatomical delineation and bone-to-soft-tissue contrast is summarized in Table 1. Inter-rater agreement is presented in Table 2, and the frequency distribution of visual grading for each fracture-prone anatomical region is illustrated in Fig. 4.

**Table 1:** Qualitative evaluation of key imaging parameters relevant to cranio-maxillofacial and oral trauma - including technical image quality and artifacts, anatomical delineation and bone-to-soft-tissue contrast in common fracture-prone anatomical regions– was performed across five different black bone and CT-like MRI protocols. Three independent observers assessed the images: observer A, a resident with 1 year of experience in craniomaxillofacial and oral surgery; observer B, a resident with 5 years of experience in the same field; and observer C, a senior radiologist with 10 years of experience. Results are reported as median values with interquartile ranges (first parentheses) and full ranges from minimum to maximum (second parentheses), using a five-point discrete visual scale (5 = most favorable, 1 = least favorable)

Median (IQR); (Minimum - Maximum)		MRI Protocol	Observer A	Observer B	Observer C	Average
Overall	**Technical Image Quality and Artifacts**	DESS SPACE STIR SPACE SPAIR VIBE-DIXON UTE	5 (1); (1–5) 4 (0); (1–5) 4 (1); (1–5) 5 (1); (2–5) 5 (0); (2–5)	5 (1); (1–5) 4 (0.75); (1–5) 4 (1); (1–5) 4.5 (1); (2–5) 5 (0); (2–5)	5 (1); (2–5) 4 (0.75); (1–5) 4 (1); (1–5) 4 (1); (2–5) 5 (0); (2–5)	5 (1); (1.33-5) 4 (0.5); (1–5) 4 (1); (1–5) 4.5 (1); (2–5) 5 (0); (2–5)
Nasoseptal	**Anatomical Delineation**	DESS SPACE STIR SPACE SPAIR VIBE-DIXON UTE	3 (1); (1–4) 4 (0); (1–5) 4 (1); (1–5) 4.5 (1); (2–5) 5 (0); (2–5)	3 (0); (1–4) 4 (0); (1–5) 4 (0); (1–5) 4 (1); (2–5) 5 (0); (2–5)	3 (1); (1–4) 4 (0); (1–5) 4 (0); (1–5) 4 (1); (2–5) 4 (0); (2–5)	3 (0.67); (1–4) 4 (0); (1–5) 4 (0.33); (1–5) 4.17 (1); (2–5) 4.67 (0); (2–5)
	**Bone-to-soft-tissue contrast**	DESS SPACE STIR SPACE SPAIR VIBE-DIXON UTE	3 (0); (1–4) 4 (0); (1–5) 4 (1); (1–4) 4 (0); (2–4) 4 (1); (2–5)	3 (0); (1–3) 4 (0.75); (1–4) 4 (1); (1–4) 4 (0); (2–4) 4 (1); (2–5)	3 (0); (1–4) 4 (0); (1–4) 4 (1); (1–4) 4 (1); (2–5) 4 (1); (2–5)	3 (0); (1-3.67) 4 (0.25); (1-4.33) 4 (1); (1–4) 4 (0.33); (2-4.33) 4 (1); (2–5)
Orbital	**Anatomical Delineation**	DESS SPACE STIR SPACE SPAIR VIBE-DIXON UTE	3 (0.75); (1–4) 3 (0.75); (1–4) 3 (0); (1–4) 5 (0); (3–5) 5 (0); (2–5)	3 (0); (1–3) 3 (0); (1–4) 3 (0); (1–3) 5 (0); (2–5) 5 (0); (2–5)	3 (0.75); (1–4) 3 (0.75); (1–4) 3 (0); (1–4) 5 (1); (2–5) 5 (0); (2–5)	3 (0.5); (1-3.67) 3 (0.5); (1–4) 3 (0); (1-3.67) 5 (0.33); (2.33-5) 5 (0); (2–5)
	**Bone-to-soft-tissue contrast**	DESS SPACE STIR SPACE SPAIR VIBE-DIXON UTE	3 (0.75); (1–3) 3 (1); (1–3) 3 (0); (1–4) 5 (1); (3–5) 4 (0.75); (2–5)	3 (1); (1–3) 3 (0.75); (1–3) 3 (0); (1–3) 5 (1); (3–5) 4 (0.75); (1–4)	2 (1); (1–3) 3 (1); (1–3) 3 (0); (1–3) 4 (1); (3–5) 4 (0); (1–4)	2.67 (0.92); (1–3) 3 (0.92); (1–3) 3 (0); (1-3.33) 4.67 (1); (3–5) 4 (0.5); (1.33–4.33)
Naso-Orbito-Ethmoidal	**Anatomical Delineation**	DESS SPACE STIR SPACE SPAIR VIBE-DIXON UTE	3 (0); (1–4) 3 (0.75); (1–5) 3 (0); (1–4) 4 (1); (2–5) 4 (0); (2–5)	3 (0); (1–4) 3 (0.75); (1–5) 3 (1); (1–5) 4 (0); (1–5) 5 (0); (2–5)	3 (0); (1–4) 3 (0.75); (1–5) 3 (0); (1–5) 4 (0); (1–5) 5 (0); (2–5)	3 (0); (1–4) 3 (0.75); (1–5) 3 (0); (1-4.67) 4 (0.33); (1.33-5) 4.67 (0); (2–5)
	**Bone-to-soft-tissue contrast**	DESS SPACE STIR SPACE SPAIR VIBE-DIXON UTE	3 (0.75); (1–4) 3 (0.75); (1–4) 3 (1); (1–4) 4 (1.5); (2–5) 4 (1); (2–5)	3 (0.75); (1–3) 3 (0.75); (1.4) 3 (1); (1–4) 4 (0.75); (1–5) 4 (1); (2–5)	2.5 (1); (1–3) 3 (0.75); (1–4) 3 (0); (1–3) 4 (0.75); (1–5) 4 (0.75); (2–5)	2.83 (0.83); (1-3.33) 3 (0.75); (1–4) 3 (0.67); (1-3.67) 4 (0.75); (1.33-5) 4 (0.92); (2–5)
Zygomaticomaxillar	**Anatomical Delineation**	DESS SPACE STIR SPACE SPAIR VIBE-DIXON UTE	2 (0); (1–3) 3 (1); (1–3) 3 (0); (1–4) 4 (0.75); (2–5) 5 (0); (2–5)	2 (1); (1–3) 3 (1); (1–3) 3 (0); (1–4) 4 (0.75); (1–5) 5 (0); (2–5)	2 (0.75); (1–3) 3 (1); (1–3) 3 (0); (1–4) 4 (1); (1–5) 5 (0); (2–5)	2 (0.58); (1–3) 3 (1); (1–3) 3 (0); (1–4) 4 (0.83); (1.33-5) 5 (0); (2–5)
	**Bone-to-soft-tissue contrast**	DESS SPACE STIR SPACE SPAIR VIBE-DIXON UTE	2 (1); (1–3) 2 (1); (1–3) 3 (1); (1–4) 4 (1.5); (2–5) 5 (1); (2–5)	2 (1); (1–3) 2 (1); (1–3) 3 (1); (1–3) 4 (1.5); (1–5) 5 (1); (2–5)	2 (0); (1–3) 2 (1); (1–3) 3 (0); (1–3) 4 (0.75); (1–5) 5 (1); (2–5)	2 (0.67); (1–3) 2 (1); (1–3) 3 (0.67); (1–3) 4 (1.25); (1.33-5) 5 (1); (2–5)
Le Fort	**Anatomical Delineation**	DESS SPACE STIR SPACE SPAIR VIBE-DIXON UTE	3 (1); (1–4) 3 (1); (1–4) 3 (0); (1–4) 4 (0); (2–5) 5 (1); (2–5)	3 (0); (1–4) 3 (0); (1–4) 3 (0); (1–4) 4 (0); (1–4) 5 (0); (2–5)	3 (1); (1–3) 3 (1); (1–4) 3 (0.75); (1–4) 4 (0); (2–4) 5 (0); (2–5)	3 (0.67); (1-3.67) 3 (0.67); (1–4) 3 (0.25); (1–4) 4 (0); (1.67–4.33) 5 (0.33); (2–5)
	**Bone-to-soft-tissue contrast**	DESS SPACE STIR SPACE SPAIR VIBE-DIXON UTE	3 (1); (1–3) 3 (0); (1–4) 3 (0); (1–4) 4 (0); (2–4) 4 (1); (2–5)	3 (1); (1–3) 3 (0); (1–4) 3 (0); (1–4) 4 (0); (1–4) 4 (1); (2–5)	3 (1); (1–3) 3 (0.75); (1–4) 3 (0); (1–3) 4 (0.75); (2–4) 4.5 (1); (2–5)	3 (1); (1–3) 3 (0.25); (1–4) 3 (0); (1-3.67) 4 (0.25); (1.67-4) 4.17 (1); (2–5)
Mandibular General	**Anatomical Delineation**	DESS SPACE STIR SPACE SPAIR VIBE-DIXON UTE	3.5 (1); (1–5) 3 (1); (1–5) 3 (1); (1–5) 5 (0); (2–5) 5 (0); (2–5)	3 (1); (1–5) 3 (1); (1–5) 3 (1); (1–5) 5 (0); (1–5) 5 (0.75); (2–5)	3 (1); (1–5) 3 (1); (1–5) 3 (1); (1–5) 5 (0); (2–5) 5 (0); (2–5)	3.16 (1); (1–5) 3 (1); (1–5) 3 (1); (1–5) 5 (0); (1.67-5) 5 (0.25); (2–5)
	**Bone-to-soft-tissue contrast**	DESS SPACE STIR SPACE SPAIR VIBE-DIXON UTE	3 (1); (1–4) 3 (1); (1–4) 3 (1); (1–4) 5 (1); (2–5) 4 (1); (2–5)	2 (1); (1–4) 2 (1); (1–4) 3 (2); (1–5) 5 (1); (1–5) 4 (1); (2–5)	2 (1); (1–4) 3 (1); (1–4) 3 (2); (1–5) 5 (0); (2–5) 4.5 (1); (2–5)	2.33 (1); (1–4) 2.67 (1); (1–4) 3 (2); (1-4.67) 4 (0.67); (1.67-5) 4.17 (1); (2–5)
TMJ/Condylar/Subcondylar	**Anatomical Delineation**	DESS SPACE STIR SPACE SPAIR VIBE-DIXON UTE	5 (0); (1–5) 2.5 (1); (1–4) 2 (1); (1–3) 4 (0.75); (2–5) 5 (1); (2–5)	5 (0); (1–5) 2 (1); (1–4) 2 (1); (1–3) 4 (0); (1–5) 5 (1); (2–5)	5 (0); (2–5) 2 (1); (1–4) 3 (1); (1–5) 4 (0); (1–5) 5 (1); (2–5)	5 (0); (1.33-5) 2.17 (1); (1–4) 2.33 (1); (1-3.67) 4 (0.25); (1.33-5) 5 (1); (2–5)
	**Bone-to-soft-tissue contrast**	DESS SPACE STIR SPACE SPAIR VIBE-DIXON UTE	5 (0); (1–5) 2 (0); (1–3) 2 (1); (1–3) 4 (0); (2–5) 4 (1); (2–5)	5 (1); (1–5) 2 (0); (1–3) 2 (1); (1–3) 4 (0.75); (1–4) 4 (1); (2–4)	5 (0); (1–5) 2 (0); (1–3) 3 (2); (1–5) 4 (0.75); (1–4) 4 (1); (2–5)	5 (0.33); (1–5) 2 (0); (1–3) 2.33 (1.33); (1-3.67) 4 (0.5); (1.33–4.33) 4 (1); (2-4.67)
Dental	**Anatomical Delineation**	DESS SPACE STIR SPACE SPAIR VIBE-DIXON UTE	3.5 (1); (1–5) 3 (1); (1–4) 2 (1); (1–3) 4 (0.75); (2–5) 5 (0); (2–5)	4 (1); (1–5) 2.5 (1); (1–4) 2 (1); (1–3) 4 (1); (1–5) 5 (0); (1–5)	4 (1); (1–5) 2.5 (1); (1–4) 2 (1); (1–3) 4 (1); (1–5) 5 (0); (1–5)	3.83 (1); (1–5) 2.67 (1); (1–4) 2 (1); (1–3) 4 (0.92); (1.33-5) 5 (0); (1.33-5)
	**Bone-to-soft-tissue contrast**	DESS SPACE STIR SPACE SPAIR VIBE-DIXON UTE	3 (1); (1–3) 2 (0.75); (1–3) 2 (0); (1–3) 3 (0); (2–5) 4.5 (1); (2–5)	3 (1); (1–3) 2 (1); (1–3) 2 (0); (1–3) 3 (0); (1–5) 4 (1); (1–5)	3 (1); (1–4) 2 (1); (1–3) 2 (0); (1–3) 3 (0); (1–5) 5 (1); (1–5)	3 (1); (1-3.33) 2 (0.92); (1–3) 2 (0); (1–3) 3 (0); (1.33-5) 4.5 (1); (1.33-5)

*DESS* Double-Echo Steady-State, *SPACE STIR* Fast Spin Echo Short-Tau Inversion Recovery, *SPACE SPAIR* Fast Spin Echo Spectral Attenuated Inversion Recovery,

*VIBE-DIXON* Volumetric Interpolated Breath-Hold Examination, *UTE* Ultrashort Echo Time

**Table 2:** Inter-rater agreement was assessed using the intraclass correlation coefficient (ICC) with corresponding 95% confidence intervals for overall technical image quality, artifact severity, anatomical delineation, and bone-to-soft-tissue contrast. Evaluation was performed by three observers (Observer A (resident; 1 year of experience), observer B (resident, 5 years), and observer C (senior,10 years) across eight fracture-prone anatomical regions per MRI protocol, representing the most common patterns of oral and maxillofacial trauma

Inter-rater agreement (ICC (95% CI))		MRI Protocol	Observer A and B	Observer B and C	Observer C and A	Average
Overall	**Technical Image Quality and Artifacts**	DESS SPACE STIR SPACE SPAIR VIBE-DIXON UTE	0.948 (0.858–0.981); *p* < 0.001 0.908 (0.757–0.967); *p* < 0.001 0.888 (0.7-0.961); *p* < 0.001 0.943 (0.845–0.98); *p* < 0.001 0.941 (0.832–0.979); *p* < 0.001	0.846 (0.615–0.943); *p* < 0.001 1 (1–1); *p* < 0.001 0.925 (0.792–0.974); *p* < 0.001 0.9 (0.739–0.964); *p* < 0.001 0.946 (0.853–0.981); *p* < 0.001	0.884 (0.669–0.96); *p* < 0.001 0.952 (0.862–0.983); *p* < 0.001 0.894 (0.715–0.963); *p* < 0.001 0.853 (0.63–0.946); *p* < 0.001 0.946 (0.853–0.981); *p* < 0.001	0.893 (0.714–0.961) 0.953 (0.873–0.983) 0.902 (0.736–0.966) 0.899 (0.738–0.963) 0.944 (0.846–0.980)
Nasoseptal	**Anatomical Delineation**	DESS SPACE STIR SPACE SPAIR VIBE-DIXON UTE	0.753 (0.425–0.906); *p* < 0.001 0.962 (0.895–0.987); *p* < 0.001 0.888 (0.699–0.961); *p* < 0.001 0.866 (0.659–0.951); *p* < 0.001 0.949 (0.925–0.991); *p* < 0.001	0.847 (0.617–0.944); *p* < 0.001 1 (1–1); *p* < 0.001 0.853 (0.763–0.973); *p* < 0.001 0.832 (0.583–0.938); *p* < 0.001 0.946 (0.921–0.990); *p* < 0.001	0.9 (0.739–0.964); *p* < 0.001 0.962 (0.895–0.987); *p* < 0.001 0.715 (0.338–0.894); *p* < 0.001 0.8 (0.517–0.925); *p* < 0.001 0.902 (0.743–0.965); *p* < 0.001	0.833 (0.593–0.938) 0.975 (0.930–0.991) 0.819 (0.6-0.943) 0.833 (0.586–0.938) 0.932 (0.863–0.982)
	**Bone-to-soft-tissue contrast**	DESS SPACE STIR SPACE SPAIR VIBE-DIXON UTE	0.846 (0.615–0.943); *p* < 0.001 0.848 (0.620–0.944); *p* < 0.001 0.896 (0.719–0.964); *p* < 0.001 0.8 (0.517–0.925); *p* < 0.001 0.790 (0.498–0.921); *p* < 0.001	0.766 (0.451–0.912); *p* < 0.001 0.907 (0.754–0.966); *p* < 0.001 0.807 (0.517–0.931); *p* < 0.001 0.764 (0.446–0.911); *p* < 0.001 0.902 (0.743–0.965); *p* < 0.001	0.618 (0.194–0.848); *p* = 0.004 0.889 (0.712–0.96); *p* < 0.001 0.807 (0.517–0.931); *p* < 0.001 0.752 (0.491–0.913); *p* < 0.001 0.788 (0.493–0.92); *p* < 0.001	0.743 (0.42–0.901) 0.881 (0.698–0.957) 0.837 (0.584–0.942) 0.772 (0.485–0.916) 0.826 (0.578–0.935)
Orbital	**Anatomical Delineation**	DESS SPACE STIR SPACE SPAIR VIBE-DIXON UTE	0.718 (0.361–0.891); *p* < 0.001 0.834 (0.589–0.939); *p* < 0.001 0.892 (0.71–0.963); *p* < 0.001 0.769 (0.34–0.919); *p* < 0.001 1 (1–1); *p* < 0.001	0.743 (0.407–0.902); *p* < 0.001 0.834 (0.589–0.939); *p* < 0.001 0.822 (0.549–0.936); *p* < 0.001 0.871 (0.671–0.953); *p* < 0.001 0.974 (0.925–0.991); *p* < 0.001	0.68 (0.381–0.844); *p* < 0.001 0.776 (0.470–0.916); *p* < 0.001 0.732 (0.368–0.901); *p* < 0.001 0.738 (0.397-0.9); *p* < 0.001 0.949 (0.861–0.982); *p* < 0.001	0.713 (0.383–0.879) 0.815 (0.549–0.931) 0.815 (0.542–0.933) 0.793 (0.469–0.924) 0.974 (0.929–0.991)
	**Bone-to-soft-tissue contrast**	DESS SPACE STIR SPACE SPAIR VIBE-DIXON UTE	0.787 (0.491–0.92); *p* < 0.001 0.670 (0.278–0.871); *p* = 0.002 0.838 (0.583–0.942); *p* < 0.001 0.92 (0.786–0.971); *p* < 0.001 0.928 (0.794–0.975); *p* < 0.001	0.787 (0.491–0.92); *p* < 0.001 0.787 (0.491–0.92); *p* < 0.001 0.8 (0.503–0.928); *p* < 0.001 0.884 (0.667–0.959); *p* < 0.001 0.951 (0.866–0.983); *p* < 0.001	0.655 (0.253–0.864); *p* = 0.002 0.586 (0.145–0.833); *p* = 0.007 0.789 (0.480–0.924); *p* < 0.001 0.850 (0.571–0.948); *p* < 0.001 0.883 (0.665–0.959); *p* < 0.001	0.743 (0.412–0.901) 0.681 (0.304–0.875) 0.809 (0.522–0.931) 0.885 (0.675–0.959) 0.921 (0.775–0.972)
Naso-Orbito-Ethmoidal	**Anatomical Delineation**	DESS SPACE STIR SPACE SPAIR VIBE-DIXON UTE	0.926 (0.802–0.974); *p* < 0.001 0.962 (0.895–0.987); *p* < 0.001 0.879 (0.679–0.958); *p* < 0.001 0.881 (0.693–0.957); *p* < 0.001 0.949 (0.861–0.982); *p* < 0.001	0.852 (0.627–0.946); *p* < 0.001 0.834 (0.589–0.939); *p* < 0.001 0.89 (0.705–0.962); *p* < 0.001 0.833 (0.587–0.938); *p* < 0.001 0.949 (0.861–0.982); *p* < 0.001	0.8 (0.517–0.925); *p* < 0.001 0.834 (0.589–0.939); *p* < 0.001 0.874 (0.666–0.956); *p* < 0.001 0.878 (0.651–0.957); *p* < 0.001 0.944 (0.84–0.98); *p* < 0.001	0.859 (0.649–0.948) 0.877 (0.691–0.955) 0.881 (0.683–0.959) 0.864 (0.644–0.951) 0.947 (0.854–0.981)
	**Bone-to-soft-tissue contrast**	DESS SPACE STIR SPACE SPAIR VIBE-DIXON UTE	0.925 (0.798–0.973); *p* < 0.001 0.787 (0.491–0.92); *p* < 0.001 0.704 (0.318–0.890); *p* < 0.001 0.898 (0.734–0.963); *p* < 0.001 0.948 (0.859–0.982); *p* < 0.001	0.736 (0.394–0.899); *p* < 0.001 0.732 (0.368–0.901); *p* < 0.001 0.790 (0.374–0.829); *p* < 0.001 1 (1–1); *p* < 0.001 0.950 (0.863–0.982); *p* < 0.001	0.834 (0.539–0.907); *p* < 0.001 0.738 (0.397-0.9); *p* < 0.001 0.744 (0.392–0.906); *p* < 0.001 0.898 (0.734–0.963); *p* < 0.001 0.899 (0.736–0.964); *p* < 0.001	0.832 (0.574–0.926) 0.752 (0.419–0.907) 0.746 (0.361–0.875) 0.932 (0.823–0.975) 0.932 (0.819–0.976)
Zygomaticomaxillar	**Anatomical Delineation**	DESS SPACE STIR SPACE SPAIR VIBE-DIXON UTE	0.8 (0.517–0.925); *p* < 0.001 0.920 (0.786–0.971); *p* < 0.001 0.870 (0.657–0.954); *p* < 0.001 0.858 (0.641–0.948); *p* < 0.001 1 (1–1); *p* < 0.001	0.901 (0.74–0.964); *p* < 0.001 0.958 (0.88–0.985); *p* < 0.001 0.806 (0.516–0.931); *p* < 0.001 0.857 (0.638–0.947); *p* < 0.001 1 (1–1); *p* < 0.001	0.781 (0.474–0.924); *p* < 0.001 0.920 (0.786–0.971); *p* < 0.001 0.928 (0.799–0.975); *p* < 0.001 0.776 (0.470–0.916); *p* < 0.001 1 (1–1); *p* < 0.001	0.827 (0.577–0.938) 2.185 (0.817–0.976) 0.868 (0.657–0.953) 0.830 (0.583–0.937) 1 (1–1)
	**Bone-to-soft-tissue contrast**	DESS SPACE STIR SPACE SPAIR VIBE-DIXON UTE	0.889 (0.682–0.961); *p* < 0.001 0.874 (0.677–0.954); *p* < 0.001 0.816 (0.52–0.937); *p* < 0.001 0.971 (0.919–0.990); *p* < 0.001 1 (1–1); *p* < 0.001	0.8 (0.517–0.925); *p* < 0.001 0.933 (0.807–0.976); *p* < 0.001 0.815 (0.423–0.941); *p* = 0.002 0.917 (0.78–0.97); *p* < 0.001 0.953 (0.871–0.983); *p* < 0.001	0.8 (0.517–0.925); *p* < 0.001 0.874 (0.677–0.954); *p* < 0.001 0.752 (0.261–0.917); *p* = 0.007 0.878 (0.687–0.956); *p* < 0.001 0.976 (0.931–0.992); *p* < 0.001	0.83 (0.572–0.937) 0.894 (0.717–0.961) 0.794 (0.401–0.932) 0.922 (0.795–0.972) 0.976 (0.934–0.992)
Le Fort	**Anatomical Delineation**	DESS SPACE STIR SPACE SPAIR VIBE-DIXON UTE	0.874 (0.677–0.954); *p* < 0.001 0.901 (0.742–0.964); *p* < 0.001 0.782 (0.482–0.918); *p* < 0.001 0.831 (0.582–0.938); *p* < 0.001 0.933 (0.809–0.977); *p* < 0.001	0.8 (0.517–0.925); *p* < 0.001 0.901 (0.742–0.964); *p* < 0.001 0.881 (0.693–0.957); *p* < 0.001 0.923 (0.795–0.972); *p* < 0.001 1 (1–1); *p* < 0.001	0.770 (0.341–0.92); *p* = 0.004 0.795 (0.507–0.923); *p* < 0.001 0.832 (0.585–0.938); *p* < 0.001 0.821 (0.560–0.933); *p* < 0.001 0.933 (0.809–0.977); *p* < 0.001	0.815 (0.512–0.933) 0.866 (0.664–0.950) 0.832 (0.587–0.938) 0.858 (0.646–0.948) 0.955 (0.873–0.985)
	**Bone-to-soft-tissue contrast**	DESS SPACE STIR SPACE SPAIR VIBE-DIXON UTE	0.749 (0.417–0.904); *p* < 0.001 0.782 (0.482–0.918); *p* < 0.001 0.851 (0.614–0.948); *p* < 0.001 0.844 (0.553–0.945); *p* < 0.001 0.946 (0.853–0.981); *p* < 0.001	0.920 (0.786–0.971); *p* < 0.001 0.881 (0.693–0.957); *p* < 0.001 0.789 (0.480–0.924); *p* < 0.001 0.866 (0.658–0.951); *p* < 0.001 0.907 (0.754–0.966); *p* < 0.001	0.832 (0.583–0.938); *p* < 0.001 0.832 (0.585–0.938); *p* < 0.001 0.789 (0.480–0.924); *p* < 0.001 0.782 (0.482–0.918); *p* < 0.001 0.928 (0.794–0.975); *p* < 0.001	0.834 (0.595–0.938) 0.832 (0.587–0.938) 0.81 (0.525–0.932) 0.831 (0.564–0.938) 0.927 (0.800-0.974)
Mandibular General	**Anatomical Delineation**	DESS SPACE STIR SPACE SPAIR VIBE-DIXON UTE	0.935 (0.824–0.977); *p* < 0.001 0.947 (0.854–0.981); *p* < 0.001 0.969 (0.911–0.990); *p* < 0.001 0.938 (0.823–0.978); *p* < 0.001 0.975 (0.928–0.991); *p* < 0.001	0.923 (0.795–0.972); *p* < 0.001 1 (1–1); *p* < 0.001 0.841 (0.590–0.943); *p* < 0.001 0.963 (0.895–0.987); *p* < 0.001 0.975 (0.928–0.991); *p* < 0.001	0.935 (0.824–0.977); *p* < 0.001 0.947 (0.854–0.981); *p* < 0.001 0.878 (0.675–0.957); *p* < 0.001 0.974 (0.861–0.982); *p* < 0.001 0.944 (0.840–0.980); *p* < 0.001	0.931 (0.814–0.975) 0.965 (0.903–0.987) 0.896 (0.725–0.963) 0.958 (0.86–0.982) 0.965 (0.899–0.987)
	**Bone-to-soft-tissue contrast**	DESS SPACE STIR SPACE SPAIR VIBE-DIXON UTE	0.925 (0.8-0.973); *p* < 0.001 0.911 (0.765–0.968); *p* < 0.001 0.898 (0.723–0.964); *p* < 0.001 0.895 (0.699–0.963); *p* < 0.001 0.949 (0.861–0.982); *p* < 0.001	0.916 (0.778–0.970); *p* < 0.001 0.937 (0.830–0.978); *p* < 0.001 0.887 (0.698–0.961); *p* < 0.001 0.904 (0.748–0.965); *p* < 0.001 0.847 (0.616–0.944); *p* < 0.001	0.840 (0.601–0.941); *p* < 0.001 0.892 (0.719–0.961); *p* < 0.001 0.862 (0.590–0.954); *p* < 0.001 0.93 (0.693–0.963); *p* < 0.001 0.907 (0.754–0.966); *p* < 0.001	0.894 (0.726–0.961) 0.913 (0.771–0.969) 0.882 (0.670–0.96) 0.91 (0.713–0.964) 0.901 (0.744–0.964)
TMJ/Condylar/Subcondylar	**Anatomical Delineation**	DESS SPACE STIR SPACE SPAIR VIBE-DIXON UTE	0.989 (0.969–0.996); *p* < 0.001 0.952 (0.869–0.983); *p* < 0.001 0.935 (0.899–0.989); *p* < 0.001 0.954 (0.766–0.968); *p* < 0.001 0.947 (0.848–0.981); *p* < 0.001	0.976 (0.932–0.992); *p* < 0.001 0.947 (0.855–0.981); *p* < 0.001 0.927 (0.781–0.975); *p* < 0.001 0.911 (0.766–0.968); *p* < 0.001 0.899 (0.737–0.964); *p* < 0.001	0.987 (0.964–0.996); *p* < 0.001 0.888 (0.71–0.959); *p* < 0.001 0.927 (0.781–0.975); *p* < 0.001 0.954 (0.766–0.968); *p* < 0.001 0.947 (0.848–0.981); *p* < 0.001	0.984 (0.955–0.995) 0.929 (0.811–0.974) 0.93 (0.82–0.98) 0.94 (0.766–0.968) 0.931 (0.811–0.975)
	**Bone-to-soft-tissue contrast**	DESS SPACE STIR SPACE SPAIR VIBE-DIXON UTE	0.96 (0.89–0.986); *p* < 0.001 0.846 (0.615–0.943); *p* < 0.001 0.863 (0.641–0.952); *p* < 0.001 0.829 (0.577–0.937); *p* < 0.001 0.842 (0.606–0.942); *p* < 0.001	0.96 (0.89–0.986); *p* < 0.001 0.917 (0.761–0.971); *p* < 0.001 0.846 (0.615–0.943); *p* < 0.001 0.906 (0.732–0.967); *p* < 0.001 0.914 (0.755–0.970); *p* < 0.001	1 (1–1); *p* < 0.001 0.846 (0.615–0.943); *p* < 0.001 0.846 (0.615–0.943); *p* < 0.001 0.829 (0.577–0.937); *p* < 0.001 0.894 (0.724–0.962); *p* < 0.001	0.973 (0.927–0.991) 0.87 (0.664–0.952) 0.852 (0.624–0.946) 0.855 (0.577–0.947) 0.883 (0.695–0.958)
Dental	**Anatomical Delineation**	DESS SPACE STIR SPACE SPAIR VIBE-DIXON UTE	0.974 (0.927–0.991); *p* < 0.001 0.910 (0.762–0.967); *p* < 0.001 0.915 (0.766–0.971); *p* < 0.001 0.940 (0.827–0.979); *p* < 0.001 0.98 (0.944–0.993); *p* < 0.001	0.951 (0.865–0.982); *p* < 0.001 0.953 (0.865–0.983); *p* < 0.001 0.915 (0.766–0.971); *p* < 0.001 0.940 (0.827–0.979); *p* < 0.001 0.962 (0.894–0.986); *p* < 0.001	0.931 (0.815–0.975); *p* < 0.001 0.953 (0.865–0.983); *p* < 0.001 0.902 (0.708–0.967); *p* < 0.001 0.940 (0.827–0.979); *p* < 0.001 0.962 (0.894–0.986); *p* < 0.001	0.952 (0.869–0.983) 0.939 (0.831–0.978) 0.911 (0.747–0.97) 0.940 (0.827–0.979) 0.968 (0.911–0.988)
	**Bone-to-soft-tissue contrast**	DESS SPACE STIR SPACE SPAIR VIBE-DIXON UTE	0.851 (0.626–0.945); *p* < 0.001 0.816 (0.472–0.936); *p* < 0.001 0.896 (0.718–0.964); *p* < 0.001 0.95 (0.865–0.982); *p* < 0.001 0.928 (0.794–0.975); *p* < 0.001	0.789 (0.496–0.921); *p* < 0.001 0.689 (0.309–0.879); *p* < 0.001 0.945 (0.836–0.981); *p* < 0.001 0.96 (0.886–0.985); *p* < 0.001 0.948 (0.859–0.982); *p* < 0.001	0.780 (0.370–0.923); *p* < 0.001 0.816 (0.472–0.936); *p* < 0.001 0.945 (0.836–0.981); *p* < 0.001 0.95 (0.865–0.982); *p* < 0.001 0.926 (0.787–0.974); *p* < 0.001	0.807 (0.497–0.93) 0.774 (0.418–0.917) 0.929 (0.797–0.975) 0.953 (0.872–0.983) 0.934 (0.813–0.977)

*DESS* Double-Echo Steady-State, *SPACE STIR* Fast Spin Echo Short-Tau Inversion Recovery, *SPACE SPAIR* Fast Spin Echo Spectral Attenuated Inversion Recovery, *VIBE-DIXON* Volumetric Interpolated Breath-Hold Examination, *UTE* Ultrashort Echo Time Inter-rater agreement is interpreted as follows: poor (< 0.5), moderate (0.5–0.75), good (0.75–0.9), and excellent (> 0.9) [27]

## Discussion

This prospective comparative study aimed to evaluate the effectiveness and feasibility of five optimized Black Bone and CT-like MRI protocols for delineating fracture-prone regions in oral and maxillofacial trauma. The findings revealed that the UTE and VIBE-DIXON sequences outperformed the other protocols in most regions, providing superior image quality, precise anatomical delineation, and enhanced bone-to-soft tissue contrast. Furthermore, the results highlight the potential advantages of radiation-free, trauma-type-specific dentomaxillofacial MRI protocols, considering the clinical reliance on imaging for managing acute, complex oral and maxillofacial trauma, and for guiding both surgical and non-surgical interventions.

MR-based depiction of the dentomaxillofacial complex is technically demanding due to the intricate anatomical configuration of dense bone, adjacent soft tissues, and nearby air-filled cavities [28]. The low proton density and short T2 relaxation time (~ 390 µs at 3 T) of bone [29] can be effectively addressed by implementing ultra-short echo times (TE), short repetition times (TR), and low flip angles [16, 30] to enhance contrast and enable rapid acquisition [16]. These techniques are increasingly investigated as complementary or stand-alone alternatives to CT in craniomaxillofacial and oral surgery-related pathologies, with applications including virtual surgical planning [31], cephalometric analysis [32], craniosynostosis assessment [33, 34], oral soft tissue tumor evaluation [35], dental implant planning [36, 37], neurovascular visualization [38, 39], and perioperative trauma diagnostics [20–22].

**Fig. 5:**
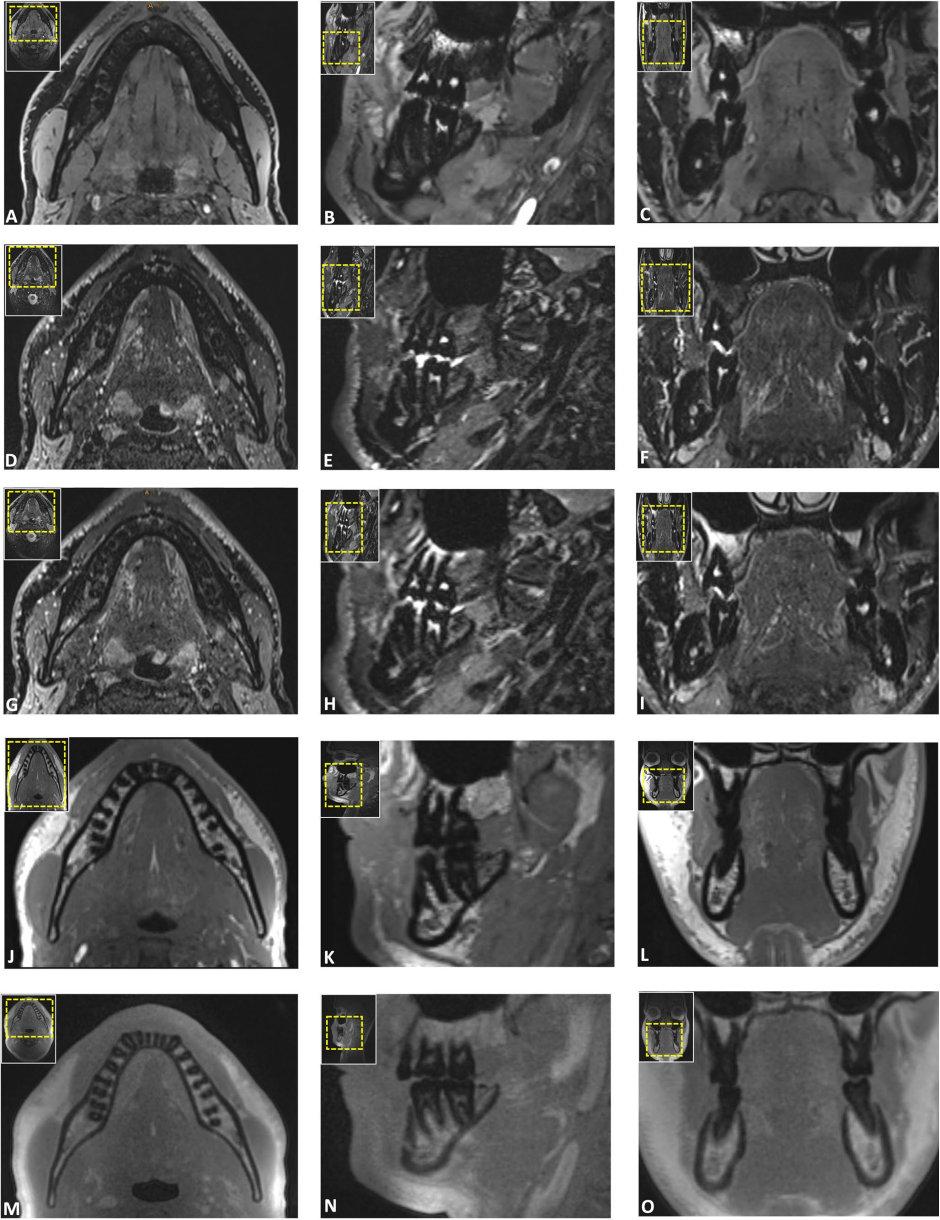
Axial, sagittal, and coronal reconstructions illustrating the mandible and dentoalveolar complex, the second most common site of facial fractures, often show fractures at two locations with potential temporomandibular joint dislocation. The following Black Bone and CT-like MRI protocols were used: T2-weighted 3D double-echo steady-state (DESS) (**A**-**C**), T2-weighted fast spin echo short-tau inversion recovery (SPACE STIR) (**D**-**F**), T2-weighted fast spin echo spectral attenuated inversion recovery (SPACE SPAIR) (**G**-**I**)) and CT-like MRI (T1-weighted volumetric interpolated breath-hold examination (VIBE-DIXON) (**J**-**L**), gradient-echo based prototype ultrashort echo time (UTE) (**M**-**O**)) MRI. The inter-protocol comparison demonstrates a clear advantage of CT-like MRI in visualizing mineralized structures, while Black Bone MRI, particularly DESS MRI, excels in soft tissue contrast, especially for visualizing peripheral nerves. Additionally, STIR and SPAIR MRI protocols are highly effective for visualizing the dental pulp

**Fig. 6:**
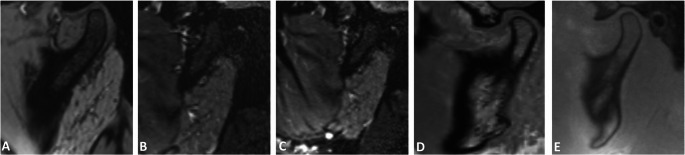
The MRI protocols used to assess the temporomandibular joint included double-echo steady-state (DESS) (**A**), fast spin echo short-tau inversion recovery (SPACE STIR) (**B**), fast spin echo spectral attenuated inversion recovery (SPACE SPAIR) (**C**), volumetric interpolated breath-hold examination (VIBE-DIXON) (**D**), and ultrashort echo time (UTE) (**M**-**O**), with particular focus on the condylar and subcondylar regions, which are common sites of trauma. Simultaneous visualization of both soft and hard tissues in conventional and dynamic MRI can offer a significant advantage for diagnostic accuracy and treatment planning

In trauma, few studies have compared STIR, DESS, T1-weighted GRE, and T1-weighted Fast Field echo (FFE) protocols at 3 T to CT for acute mandibular fractures in young adults [21] and older patients [20, 22], assessing diagnostic performance of CT-like MRI [21], associated inferior alveolar nerve impairment [20], and severity of mandibular fracture dislocation between the mandibular and mental foramina [22]. Burian et al. reported the highest image quality and lowest artifact susceptibility with T1-FFE (4.2/5), followed by STIR (4.0/5) and DESS (3.9/5), with good to excellent inter-rater agreement (ICCs: 0.792–0.865) [20]. Feuerriegel et al. confirmed these findings for T1-GRE, reporting high image quality (4.3 ± 0.6) and bone-to-soft-tissue contrast (4.7 ± 0.44), rated on a 5-point scale, along with excellent inter-rater agreement (κ = 0.86) [21]. Our findings confirm that VIBE-DIXON and UTE provided the best mandibular delineation, with VIBE-DIXON achieving the highest scores (median: 5, IQR: 0) and excellent inter-rater agreement (ICC = 0.944–0.974; all *p* < 0.001), and outperforming UTE in bone-to-soft-tissue contrast (5, IQR: 0 vs. 4.5, IQR: 1) (Fig. 5). The DESS protocol, while generally lower in performance compared to the UTE and VIBE-DIXON protocols, demonstrated particular strength in delineating trauma-prone condylar regions involving the TMJ with its superior bone-to-soft tissue contrast, which can be critical for preoperative planning and surgical intervention (Fig. 6). In trauma settings, UTE, ZTE, and T1-FFE provided high-resolution bone detail, STIR was best for edema, and DESS for anatomical nerve depiction [20, 21]– findings reflected in our region-specific visual grading. Additionally, CT-like MRI matched CT in fracture detection, classification, and dislocation assessment, while simultaneously allowing for evaluation of inferior alveolar nerve impairment [20–22].

**Fig. 7:**
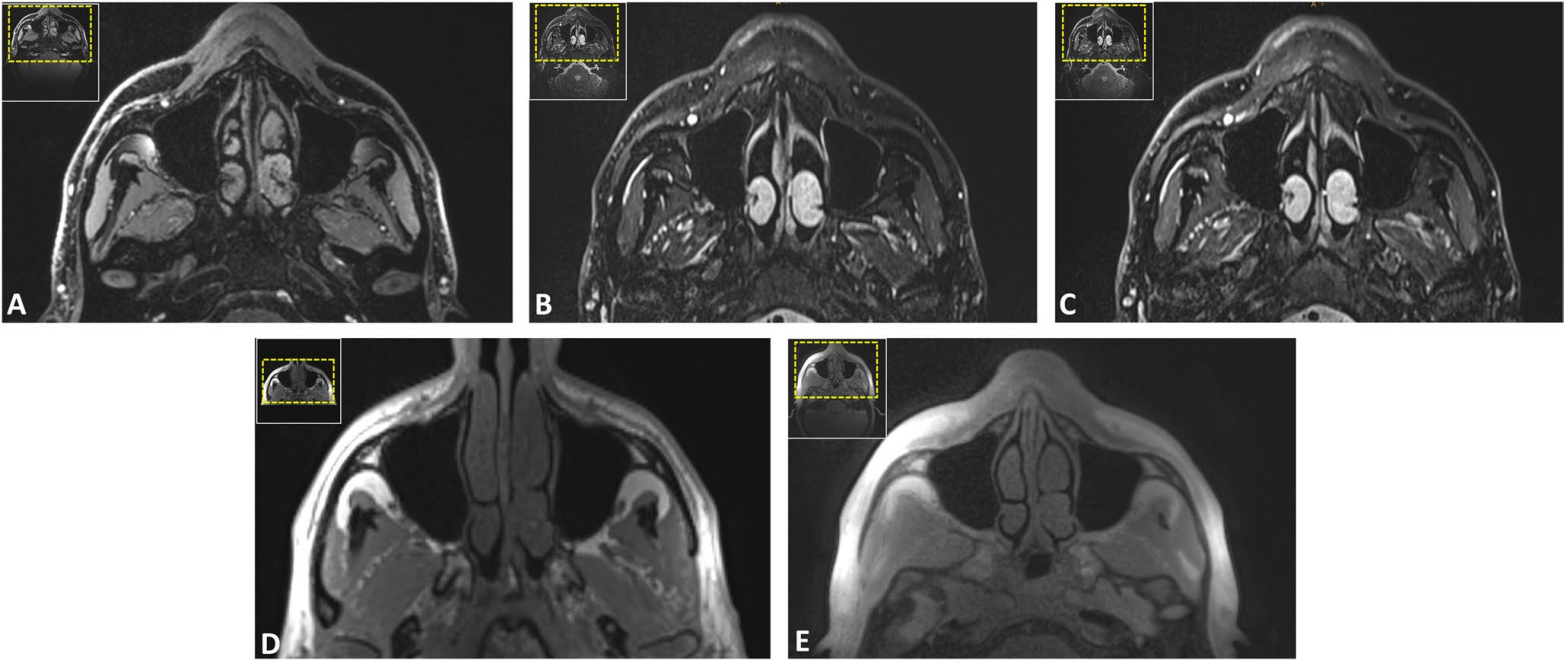
Axial reconstructions illustrating the delineation of the zygomaticomaxillary complex, a region frequently affected in maxillofacial trauma, where the zygoma is separated from the rest of the skull. Images were acquired using T2-weighted 3D double-echo steady-state (DESS) (**A**), T2-weighted fast spin echo short-tau inversion recovery (SPACE STIR) (**B**), T2-weighted fast spin echo spectral attenuated inversion recovery (SPACE-SPAIR) (**C**), T1-weighted volumetric interpolated breath-hold examination (VIBE-DIXON) (**D**), and gradient-echo based prototype ultrashort echo time (UTE) (**E**) MRI

No trauma studies have yet focused on the upper or mid-to-lower face. However, our results show that UTE and VIBE-DIXON protocols achieved the highest scores for image quality with minimal artifacts, excelling in visualizing nasoseptal, orbital, Le Fort, and NOE fracture-prone regions, highlighting MRI’s value for comprehensive simultaneous bone and soft tissue assessment in maxillofacial trauma [28]. These findings align with prior evidence that UTE sequences offer high-resolution, low-artifact bone visualization, which are common challenges in maxillofacial imaging [40, 41]. The enhanced bone-to-soft-tissue contrast primarily results from the use of a low flip angle, which effectively suppresses both fat and water signals, thereby enhancing bone contrast in patho-anatomical conditions where bone is surrounded by soft tissue, such as the craniomaxillofacial area [16]. A key limitation of the technique is its reduced performance at trauma sites involving air-bone-soft-tissue interfaces, such as the craniofacial sinuses, where low signals are obtained from both bone and air [16, 28]. This makes it challenging to distinguish patho-anatomical features that may be relevant in trauma-related decision-making. This was evident in our results, especially in the SPACE-based (STIR and SPAIR) and DESS sequences (Fig. 7). This limitation could be particularly clinically evident in the MRI-based detection of fine zygomaticomaxillary or NOE fractures and posterior orbital trauma, where the bone-air interface complicates visualization, potentially leading to underdiagnosis or delayed decision-making. For dental trauma delineation, several sequences demonstrated promising diagnostic potential. Our results indicate that VIBE-DIXON and UTE are particularly effective for visualizing the entire tooth structure, aligning with previously published findings. In contrast, the visualization of the dental pulp showed slight differences between sequences, with both fat-saturated SPACE sequences (STIR and SPAIR) and DESS achieving favorable results [24]. Clinically, the varying degrees of fat saturation in the SPACE sequences could be valuable for assessing pulp vitality in different types of traumatic dental injuries [42]. Inter-rater agreement was consistently high across all five protocols, highlighting the reproducibility and reliability of the selected MRI protocols. This demonstrates that dentomaxillofacial MRI, when optimized with tailored protocols and coils, has the potential to ensure consistent diagnostic outcomes in trauma settings regardless of observer experience or specialization.

Although the primary focus of this study was trauma assessment, it is important to acknowledge the distinct strengths and limitations of each MRI protocol within the broader clinical context of comprehensive head and neck imaging. STIR and SPAIR MRI provide effective fat suppression and exhibit high sensitivity for detecting edema and inflammation, thereby facilitating soft-tissue evaluation [43]. In contrast, UTE and VIBE-DIXON sequences excel at reducing metal artifacts and offer superior visualization of cortical bone and metallic implants, which is particularly valuable in postoperative imaging [40]. Additionally, the DIXON technique delivers high-quality images with enhanced soft tissue contrast, especially when combined with contrast agents, making it a promising tool for monitoring various pathologies that require long-term follow-up. Thus, by proactively considering potential postoperative evaluations, long-term disease monitoring, and complex differential diagnoses, clinicians and radiologists can optimize imaging protocols to maximize diagnostic yield and patient care outcomes.

Despite the benefits of radiation-free imaging for this radiosensitive, relatively young cohort and the superior soft-tissue contrast compared to CT, several limitations should be noted. First, MRI is not standard in emergency settings, due to its limited availability, higher costs, longer acquisition times, and reliance on patient compliance, which can present a significant drawback for fast decision-making in critical situations. Furthermore, although Black Bone and CT-like MRI techniques showed promising results, CT will still be preferred in specific clinical scenarios, where visualizing intricate bone details is necessary. Thus, the results obtained in this feasibility study should be directly compared with CT in future cross-modality studies to better assess their clinical value in relevant patient cohorts. Second, since this study was conducted only in healthy volunteers aged 18 and older, its findings cannot be extrapolated to trauma patients across all age groups, requiring further validation in a broader clinical population. Third, variability in data acquisition and scan parameters across studies limits comparability, highlighting the need for future research to standardize radiation-free MRI workflows tailored to specific indications, while clearly defining the benefits and limitations of each protocol.

## Conclusion

From a clinical perspective, the increasing evidence regarding the potential of MRI-based trauma delineation in craniomaxillofacial and oral surgery has been further confirmed. However, based on current evidence, MRI remains a complementary tool to conventional CT in the trauma setting. Nevertheless, Black Bone and CT-like MRI protocols hold significant potential to serve as a radiation-free, standalone modality with an improved benefit-to-risk ratio in indicated clinical cases that require repeated imaging, particularly for young adults and pediatric patients. As demonstrated in this study, achieving this transition requires a comprehensive understanding of the specific strengths and limitations of each MRI protocol for targeting particular anatomical areas. This highlights the importance of selecting the appropriate imaging modality, scan protocol, and coil tailored to the indication-specific diagnostic and surgical requirements of each clinical scenario, in order to optimize outcomes in personalized management of craniomaxillofacial and oral trauma.

## Data Availability

No datasets were generated or analysed during the current study.
